# Impact of maternal microbiota imbalance during pregnancy on fetal cerebral neurodevelopment: Is there a link to certain autistic disorders?

**DOI:** 10.1016/j.bbih.2025.101074

**Published:** 2025-07-28

**Authors:** Sylvie Mavel, Léa Pellé, Christian R. Andres

**Affiliations:** a UMR 1253, iBrain, Université de Tours, Inserm, 37000, Tours, France; b Service de Biochimie et de Biologie Moléculaire, CHRU de Tours, 37000, Tours, France

**Keywords:** Maternal microbiome, Fetal neurodevelopment, MIA

## Abstract

Autism spectrum disorder (ASD) is a neurodevelopmental disorder associated with a heterogeneous group of psychiatric disorders primarily characterized by impairments in communication and social interactions. ASD typically emerges around 18 months of age. While no single etiology has been identified, theories suggest a combination of genetic predisposition and environmental factors, such as exposure to toxics, viral infections, and neuroinflammatory processes. However, the mechanisms linking environmental risk factors to ASD remain poorly understood, particularly during the prenatal period. Among the various hypotheses, the gut microbiota has been proposed as a potential modulator of nervous system development and function. During pregnancy, the maternal microbiota could trigger gestational inflammatory responses, leading to maternal immune activation (MIA). These deleterious processes could play a causal role in the etiopathogenesis of neurodevelopmental disorders in offspring. The gut microbiota could be the missing link between genetic susceptibility and environmental exposures in certain forms of ASD. The gut microbiome induces the production of microbiota-derived signaling metabolites, immune mediators, gut hormones, and neural signaling via the spinal cord and vagus nerve. This review synthesizes the current knowledge from preclinical rodent models and human studies that investigate the impact of the maternal gut microbiota during pregnancy on ASD risk in offspring. Additionally, the potential roles of the maternal oral and vaginal microbiota are also discussed in the context of this maternal-offspring pairing. Finally, we examine how restoring maternal microbiome balance, through interventions such as pre/probiotics, may help reduce the perinatal risk of certain ASD by positively influencing prenatal environmental factors.

## Highlights

• Environmental maternal factors influence the ethology of ASD.
• Dysbiotic microbiota induces inflammation and/or dysregulated immune response.
• Maternal immune activation during pregnancy can contribute to ASD development.
• Maternal gut microbiota could impact the offsping neurodevelopment in preclinical models.

## Graphical abstract

**Figure:**
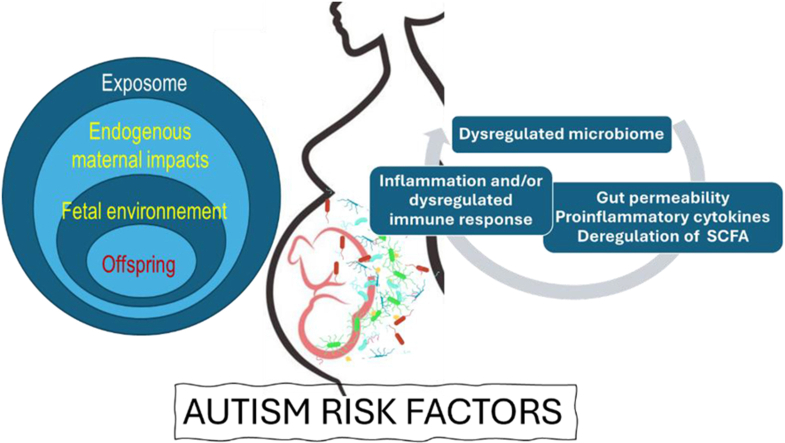

## 1 Introduction

According to the World Health Organization, autism spectrum disorders (ASD) encompass a diverse range of conditions characterized primarily by difficulties in social interaction and communication, along with restricted or repetitive patterns of behaviors, impairing the individual's ability to process and respond to external stimuli. A reliable diagnosis can be made as early as 18 months, with the mean age of diagnosis around 43 months (van ’t Hof et al., 2021).

It is estimated that approximately one in 100 children worldwide is affected by ASD, which spans all social classes (Zeidan et al., 2022). Given its prevalence and the associated societal costs, autism is recognized as a major public health concern.

It is widely accepted that ASD has multifactorial origins, with significant heterogeneity. The main hypotheses suggest genetic predispositions, with heritability for ASD estimated between 70 and 90 % (Genovese and Butler, 2023). However, these genetic factors may partly interact with various environmental factors, such as inflammation, viral infections, and toxics, particularly early development. These influences may act through several mechanisms, including epigenetic regulation.

Among the various hypotheses regarding the etiology of ASD, the role of the gut microbiota in central nervous system (CNS) function appears to be important. The gut microbiota might represent a missing link between genetics and environmental factors, as is shaped by both (Parizadeh and Arrieta, 2023).

While many reviews compile evidence of the involvement of gut microbiota dysregulation in brain dysfunction in patients with ASD (El Mazouri et al., 2024; Levkova et al., 2023; Tao et al., 2025), fewer ((Dawson et al., 2021; Sun et al., 2023) have focused on the prenatal period, a critical window for neurodevelopment, especially considering that ASD originates early in life. This raises key question: what is the impact of maternal microbiota on fetal brain development?

This review synthesizes clinical and preclinical studies on the impact of maternal microbiota on fetal neurodevelopment and its implications on “placenta-fetal brain” axis during pregnancy and its potential role in shaping the risk of neurodevelopmental disorders such as ASD in offspring.

## 2 Gut microbiota and autism

### 2.1 Definition

The human gut microbiota comprises approximately 10^14^ microorganisms, including bacteria distributed across hundreds of dominant species per individual, along with other microorganisms, such as viruses, fungi, and yeasts. The gut microbiota is unique to each individual and begins forming at birth. This ecosystem is influenced by factors such as prematurity, mode of delivery, and breastfeeding (Caprara et al., 2024).

**Fig. 1:**
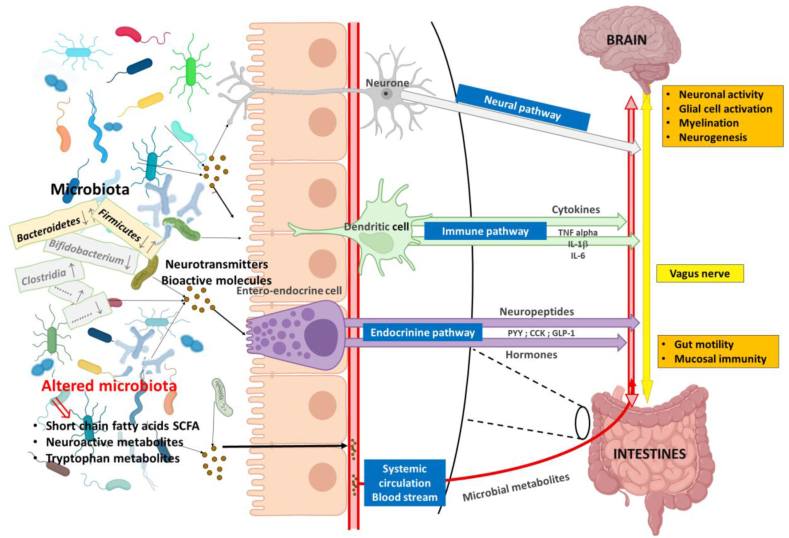
Different communication pathways of the gut-brain axis, in ASD pathology. Part of the figure was designed using resources from Biorender.com.

The « gut-brain axis » refers to the bidirectional biochemical communication between the gut microbiota and the CNS. This interaction occurs via multiple pathways, including the bloodstream, the autonomic nervous system, the enteric nervous system, the hypothalamic-pituitary-adrenal (HPA) axis, and especially the vagus nerve, the primary conduit linking the main connection between the gut and the brain (Fig. 1).

The gut microbiota plays a crucial role for host physiology, including nutrient metabolism (e.g. breakdown of fibers, proteins, and carbohydrates), maintenance of the mucosal barrier (through increased mucin production), regulation of the intestinal motility, development of the immune and metabolic systems, and, protection against pathogens (Rusch et al., 2023). Gut bacteria also produces various vitamins, amino acids, and contribute to the bile acid metabolism.

During early life, the gut microbiota is less diverse and more variable than in adulthood. Infants typically show a higher abundance of *Proteobacteria* and *Actinobacteria*. After maturation and stabilization, the gut ecosystem becomes dominated by Bacteroidetes and Firmicutes phyla (Suárez-Martínez et al., 2023). This early microbial colonization is increasingly recognized as critical for neurodevelopmental neurogenesis, myelination, glial cell function, and regulation of blood-brain barrier (BBB) permeability (Heiss and Olofsson, 2019).

### 2.2 Altered gut microbiota in patients with ASD

Gastrointestinal troubles are highly prevalent in children with ASD with rates ranging from 46 % to 84 % and, include manifestations such as constipation and diarrhea (Al-Beltagi et al., 2023). Many patients with ASD present a dysbiotic gut microbiota, characterized by reduced bacterial diversity and a lower abundance of beneficial bacteria.

A meta-analysis has shown that an unbalanced (either increased or decreased levels) of the genera *Bacteroides*, *Bifidobacterium*, *Clostridium*, *Coprococcus*, *Faecalibacterium*, *Lachnospira*, *Prevotella*, *Ruminococcus*, *Streptococcus*, and *Blautia* may be linked to ASD etiology (Levkova et al., 2023).

Another meta-analysis reports inconsistent results, but have noted dysregulations at the phylum level of Firmicutes, Bacteroidetes and Proteobacteria (El Mazouri et al., 2024). There appears to be no strong evidence of differences in α-diversity between some ASD patients and typical controls, whereas studies have consistently reported differences in β-diversity, highlighting an enrichment of pro-inflammatory genera, a reduction in specific probiotics and lactic acid-producing bacteria, and an imbalance in anti-inflammatory butyrate-producing bacteria (Tao et al., 2025).

But, in parallel, a study by Yapp and colleagues in Australia, involving 247 children (99 with ASD), found no reliable associations between ASD diagnosis and the gut microbiome. The authors suggested that autism-related dietary preferences might mediate the ASD-microbiome (Yap et al., 2021).

So, the question of whether the gut microbiome is a cause or a consequence of ASD remains open. According to Ozcan and Hsiao, it is likely both (Ozcan and Hsiao, 2022). Therefore, to improve the understanding of potential causes, clinical and preclinical studies are needed to determine whether there is a link between the maternal microbiome and the gut dysbiosis observed in certain patients with ASD. In some cases, the origin of a dysbiotic microbiome could, result from mother-to-infant microbiota transmission and could, years later, reflect the impact of this imbalance on neurodevelopment.

### 2.3 Alteration of the gastrointestinal and blood-brain barriers

**Fig. 2:**
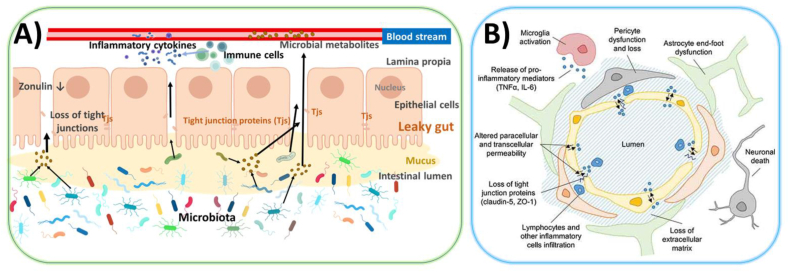
**A)** Schematic illustration of leaky gut following dysbiosis, showing increased barrier permeability (bacteria, microbial metabolites, release of lipopolysaccharides (LPS) and peptidoglycans (PGNs), etc.) which induce inflammation (increased pro-inflammatory macrophage phenotype and elevated levels of pro-inflammatory cytokines). Visualization of Zonulin, a protein that regulates the tight junctions (TJs) between cells in the intestinal wall. **B)** Schematic representation of the blood-brain barrier (BBB) in (a) physiological and (b) pathological states created by Parker et al. (Parker et al., 2019).

In the colon, one of the primary mechanisms protecting against the development of pathogenic microbiota is the two-layered mucus (Fig. 2A). Furthermore, the gut microbiota also contributes the regulation of epithelial permeability by modulating tight junctions (TJs) proteins. Abnormal intestinal permeability, often referred as “leaky gut”, has been observed in children with ASD. Elevated levels of intestinal Fatty Acid Binding Protein (I-FABP), a marker of epithelial damage, were correlated with more severe deficits in communication and social interaction as well as maladaptive behaviors, in a cohort of children with ASD aged 2–5 years (Teskey et al., 2021).

Zonulin, a modulator of TJ integrity (Fig. 2A), could serve as a peripheral marker of abnormal intestinal permeability as observed for patients with ASD (Karagözlü et al., 2022). Zonulin also affects the BBB by influencing the sealing of endothelial cell junctions. Increased permeability of both the gut and brain barriers could facilitate the translocation of blood-borne immune cells, and microbial metabolites into the CNS, potentially activating microglia via inflammatory cytokines (Fig. 2B). These processes may lead to neuro-inflammation, neuronal cell dysfunction and ultimately cell death.

### 2.4 Inflammation and immune dysregulation

As mentioned above (Fig. 1), the activation of microglia via cytokines and microbial co-metabolites contributes to neuroinflammatory state. During fetal cerebral elevated levels of pro-inflammatory cytokines can disrupt neurogenesis, neuronal migration, and synaptic plasticity through effector cells of adaptive immunity (Th1, Th2 Treg, and B cells), potentially influencing ASD pathogenesis (Robinson-Agramonte et al., 2022). However, a study conducted in Sweden (involving 318 mothers of ASD-cases and 429 mothers of unaffected controls), found no consistent association between early pregnancy maternal cytokine levels and ASD diagnosis (Brynge et al., 2022).

Microglia are influenced by both local CNS signals and peripheral input from the gut. A dysbiotic microbiome could dysregulate microglial maturation and function. However, abnormal production of pro-inflammatory cytokines (TNF-α, IL-4, IL-21, and BAFF) have been found up-regulated in some cerebrospinal fluid (CSF) of patients with ASD. This suggests a self-sustaining cycle of neuroinflammation, characterized by permanent status by persistent microglial activation, altered dendritic spine, and disrupted neuronal connectivity (Than et al., 2023).

### 2.5 Metabolic alterations

The gut microbiota transforms dietary fibers into short-chain fatty acids (SCFAs) such as acetate, propionate (PPA), and butyrate (BT) which can enter the bloodstream and cross the BBB. PPA, a major SCFA associated with ASD, is correlated with bacteria such as *Clostridia, Bacteroides,* and *Desulfovibrio* (Lagod and Naser, 2023). BT, produced, for example, by *Lactobacilli*, serves as an inhibitor of histone deacetylase (HDAC), regulating gut permeability and the BBB, and remodeling chromatin towards an open and transcriptionally active state. BT also exerts anti-inflammatory effects in the gut by modulating macrophages activity. SCFAs may modify mitochondrial function, particularly in the citric acid cycle and carnitine metabolism, or through the epigenetic modulation of genes related to ASD, via chromatin remodeling, including brain (Abdalkareem Jasim et al., 2022).

Additionally, gut microbes produce a range of neuroactive compounds and metabolites, such as γ-aminobutyrate (GABA), glutamate, oxytocin, serotonin, as well as 5- aminovalerate, taurine, *p*-cresol and bile acid metabolites (Flynn et al., 2025; Morais et al., 2021). After absorption, some of these metabolites can enter the circulatory system, eventually, cross the BBB and reach the brain, where they may play active roles, in influencing behavior later in life.

## 3 Maternal gut microbiota and neurodevelopment

When women become pregnant, their intestinal environment, as well as the vaginal, and oral microbiomes, change to adapt to pregnancy. These shifts support maternal and fetal health but may also introduce vulnerabilities.

Preclinical studies in rodents focusing on the prenatal stage, have suggested that several maternal prenatal factors (such as pesticide exposure, pollution, maternal infections (particularly *Clostridium difficile*), antibiotic use, maternal obesity, inflammation, and increased intestinal permeability) may induce or increase the risk of ASD (for review, see (Love et al., 2024)). All these processes could disrupt what is now hypothesized as the maternal gut - placenta - fetal brain axis (Sajdel-Sulkowska, 2023).

The maternal microbiota modulates microglial activity and immunity in the fetal CNS, impacting neurogenesis (Zawadzka et al., 2021). Indeed, colonization of pregnant mice with genetically modified *Escherichia coli HA107* has shown that the maternal microbiota shapes the offspring's immune system.

Before acquiring its own microbiota, the fetus is exposed to metabolites from the maternal microbiota. For example, bacterial extracellular vesicles (BEVs) (membrane-bound structures used by microbes for communication) have been detected in amniotic fluid of healthy pregnant women, resembling those found of the maternal gut (Kaisanlahti et al., 2023). In mice, these vesicals can reach the amniotic cavity and may play a critical role in initiating the prenatal immune system and facilitating postnatal gut colonization (Kaisanlahti et al., 2023). The authors also mention that the potential contribution of bacterial EVs from other sources, such as the oral or vaginal microbiota, may be involved in the mechanism of preparing the fetal immune system for early gut colonization at birth.

At birth, the meconium microbiota mostly starts developing during delivery, and infants are colonized. A study shown that immediate perinatal factors (exposure to antibiotics during birth, delivery mode) affected the composition of the meconium microbiota more than prenatal factors (maternal characteristics, environmental factors during pregnancy) (Turunen et al., 2024). However, under certain conditions, infants may be colonized before birth, challenging the “sterile womb” paradigm. For instance, some studies have described similar composition of meconium between vaginal and cesarean deliveries (Cheddadi et al., 2023). Furthermore, studies suggest that the placenta microbiome could originate from the oral or vaginal cavities (Saadaoui et al., 2024). In a germ-free mouse model, genetically tagged bacteria from the mother's intestine were detected in the fetal meconium collected after a cesarean section, supporting the possibility of bacterial transmission via amniotic fluid consumption (Vallès and Francino, 2018). Notably, over 50 % of meconium bacteria also colonized amniotic fluid, with little overlap with maternal vaginal and oral microbiota. Moreover, some bacterial species were found in umbilical cord blood, suggesting a transfer of the maternal microbiota to the fetus under certain conditions (Park et al., 2023; Srikantha and Mohajeri, 2019).

Despite these findings, many researchers remain skeptical, citing potential sample contamination (during sample collection or analysis), given the low bacterial biomass analyzed (Turunen et al., 2024). New technologies such as those employing type IIB restriction enzyme-based site-associated DNA sequencing for microbiome (2bRAD-M), offer a reduced metagenomics method for profiling species-level microbial composition, compared to the commonly used 16S rRNA amplicon sequencing and the gold-standard whole metagenomics sequencing (WMS) (Hou et al., 2025). These news methodologies may help validate or refute the sterile womb theory.

Maternal influence, through maternal bacterial dysbiosis, is the primary cause of changes in the composition and function of the intestinal microbiota in offspring (Nyangahu and Jaspan, 2019). Conversely, the brain primarily affects gastrointestinal functions via the autonomic nervous system (ANS), which can modulate motility, intestinal barriers integrity, and immune responses. This, in turn, increases stress-related pathways involving corticotropin-releasing hormone (CRH), which induces the secretion of cortisol (a stress hormone), impact intestinal immune and barrier functions, exhibiting psychotropic effects (Góralczyk-Bińkowska et al., 2022).

### 3.1 Preclinical insights

To comprehend the intricate impact of the gut microbiome on the social behaviors observed in ASD, rodent models have been employed to elucidate the alteration of brain neuronal circuits (Tartaglione et al., 2023), particularly during the prenatal stage, which is crucial for social development.

Based on a germ free (GF) pregnant mice, the absence of the maternal gut microbiome impaired thalamocortical axonogenesis in fetuses, possibly through disturbed microbial metabolites like trimethyl-*N*-oxide (TMAO), trimethyl-5-aminovalerate, and hippurate (Vuong et al., 2020). The influence of maternal gut microbiota on microglial properties (altered microglia maturation and CNS resident immune cells during fetal development), through a gender-specific pathway, was also described (Thion et al., 2018).

Transplantation of altered maternal microbiota to GF mice transmitted stem cell phenotypes to the recipient offspring, via the mTOR pathway and SCFA dysregulation (Dang et al., 2025).

Valproic acid or its salt, sodium valproate (VPA), is an antiepileptic drug that inhibits histone deacetylases, but this drug may induce ASD (Tartaglione et al., 2023). The **VPA-induced** rodent model is widely used as a preclinical model to investigate the pathophysiology of autism. It could be noticed that VPA exposure also results in alterations in the maternal gut microbiota.

**Maternal Immune Activation (MIA)** model is a ASD-related animal model from prenatal infection (Tartaglione et al., 2023), inducing changes in the gut microbiota of pregnant mice, linked to IL-17A (Kim et al., 2022). Specifically, segmented filamentous bacteria or other Th17 cell-inducing bacteria (promoting an IL-17 response) in the maternal gut favored MIA-induced pathogenesis (Kwon et al., 2022). Furthermore, some bacteria (such as *Bifidobacterium bifidum* or bacterial metabolites [e.g., SCFAs or secondary bile acids]) not only enhance Th17 cell responses but also likely mitigate their activity by augmenting anti-inflammatory Treg pathways (Kwon et al., 2022). One hypothesis proposed by the authors is that a deregulation of the Treg pathway during pregnancy could trigger MIA, potentially leading to neural dysfunction which could result in psychiatric disability.

A **maternal high-fat diet (MHFD)** has also induced a shift in microbiota composition that has negatively impacted the social behavior of offspring compared to mothers on a regular diet (Di Gesù et al., 2022). MHFD significantly impaired the intestinal mucus barrier of offspring (Sun et al., 2025b), and upregulated the tryptophan-kynurenine (Trp-Kyn) pathway in both maternal circulation and the fetal forebrain (Sun et al., 2025a). The irregular accumulation of neuroexcitatory Kyn metabolites in embryonic brain tissue caused disrupted cellular redox reactions. These mechanisms could partly explain the increased risk of ASD prevalence in children of mothers with obesity. It could be noticed that omega-3 polyunsaturated fatty acids (PUFAs) like docosahexaenoic acid (DHA) and eicosapentaenoic acid (EPA) influence positively, in preclinical studies, microglial activity, neuroinflammatory regulation, and synaptic plasticity (Al-Ewaidat et al., 2025).

Furthermore, in a mouse model, parental high salt diet was associated with ASD-like behavioral in offspring, correlated with gut dysbiosis (primarily a decrease in *Lactobacillus*) and increased intestinal permeability via pro-inflammatory Th17 responses (Afroz and Alvina, 2019).

These various dietary aspects (fats, salt, …), highlighted by preclinical studies, are still far from being extrapolated to humans, both in terms of the targeted population, the required quantities, and the potential synergistic effects between different nutrients.

All these positive associations between environmental risks factors and intestinal damage observed in rodents show that a maternal gut microbiota dysbiosis during gestation can lead to MIA, disrupt the intestinal barrier, and alter the production of key metabolites, resulting in systemic inflammation that may compromise fetal neurodevelopment.

### 3.2 Human evidence

#### 3.2.1 Exposomes

The consumption of **certain foods** by pregnant women, can modify their gut microbiota, with potential long-term effects on fetal development.

For instance, a high-fat diet establishes a pro-inflammatory profile that may promote certain abnormalities and infections (Maldonado-Ruiz et al., 2019). However, in California, USA, levels of PUFAs were measured in prospectively collected mid-pregnancy samples (between 2010 and 2011; n = 499 children with ASD versus n = 502 controls) without any strong association between maternal PUFA levels and ASD in children (Lyall et al., 2020). Despite this, the role of lipids in ASD etiology, particularly in the context of gestational obesity and inflammatory pathways, may still be of crucial importance.

The impact of soy intake also remains controversial, especially considering phytoestrogens such as genistein and daidzein, which can alter gut microbial species (they can bind to, and activate, estrogen receptors in brain) and could potentially lead to fetal neurological disorders. Furthermore, bacterial metabolites (such as equol) and virulence factors, depending on the host's ethnic origin, may induce epigenetic modifications that alter neurobehavioral responses (Rosenfeld, 2019), although this is debated.

Indeed, a large cohort (combining Norwegian and English data) based on self-reported information about the overall prenatal diet was analyzed alongside diagnostic data on the degree of autism in children, a decade after their birth. A healthy prenatal diet was associated with a 22 % reduction in ASD risk, overall the impact of maternal prenatal diet on ASD in children appears to be modest (Friel et al., 2024). Longitudinal and standardized studies are still necessary.

Many **medications** are believed to influence the composition of the microbiota, depleting beneficial species, and causing adverse effects, thereby leading to dysbiosis (Algavi and Borenstein, 2023). However, few studies focus on the prenatal effects of these medications. Specifically, antibiotics alter the microbiome through bactericidal and/or bacteriostatic actions, affecting the richness and diversity of the intestinal microbiota. Antibiotics are frequently prescribed during pregnancy (Kuperman and Koren, 2016). Meta-analyses highlight conflicting data and do not conclusively link early-life antibiotic exposure to subsequent ASD development (Fish-Williamson et al., 2022). When maternal antibiotic use was associated with an increased risk of autism, it depended on the exposure period and antibiotic classes (Njotto et al., 2023). In particular, the risk may only be increased with antibiotic exposure after 34 weeks of gestation (Lin et al., 2023). More importantly, MIA may play a larger role than the effects of antibiotics. In a study involving 116 children with ASD and 860 controls, maternal flu during the second trimester was associated with an increased risk of ASD, but only among women who did not take antibiotics during pregnancy (Holingue et al., 2020). This suggests that the incidence of autism could be reduced by 12 %–17 % if maternal infections could be prevented or safely treated in a timely manner (Tioleco et al., 2021). Valproate, an antiepileptic medication, is associated with an increased risk of ASD, as mentioned above. When multiple medications are considered, proton-pump inhibitors, metformin, antibiotics, and laxatives demonstrate the strongest associations with a disturbed microbiome (Vich Vila et al., 2020). SSRI antidepressants increase the abundance of *Eubacterium ramulus*, while tricyclic antidepressants increase the abundance of *Clostridium leptum*. Proton pump inhibitors alter the composition and diversity of the gut microbiota, reducing *Firmiculites* and increasing *Bacteroides*, along with an elevated incidence of *Clostridium difficile* (Le Bastard et al., 2018). Opioids, statins, antipsychotics, and non-steroidal anti-inflammatory drugs (NSAIDs) also alter the composition of the mother's gut microbiota (Codagnone et al., 2019). However, in a cohort study of 411 251 mother-child pairs for ASD from Hong Kong, with a delivery date between January 2001 and January 2015, there was no increased risk of ASD associated with prenatal use of antipsychotics (Wang et al., 2021).

Prenatal exposure to **environmental toxic** substances can impact the metabolic pathways of lipids, amino acids, and nucleic acids, primarily associated with energy metabolism, hormonal metabolism, oxidative stress, and inflammation (Dai et al., 2020). Environmental pollution (fine particulate matter) could increase the production of mitochondrial reactive oxygen species, an increase in pro-inflammatory oxidative lipids and the release of inflammatory cytokines, associated with an overall increase in intestinal permeability, as observed in culture cells or animal models (Filardo et al., 2022).

Results regarding the impact of food contaminants and additives on gut microbiota, such as phthalates, bisphenol A and heavy metals, suggest that they could disturb the gut microbiota and be detrimental to health (Singh et al., 2022).

A relationship between exposure to agricultural pesticides and ASD, especially during pregnancy and the early years has been highlighted (Román et al., 2024). Simultaneously, the impact of pesticides (insecticides, fungicides, and herbicides) on the gut microbiome has also been studied (for a review, see (Yang et al., 2023)). For example, one contribution of pesticide-induced dysbiosis could be an increase in brain soluble epoxide hydrolase activity, leading to a higher risk of ASD in children. Furthermore, it has been suggested that reactive oxygen species (ROS) and prostaglandin E2 synthesis, acetylcholinesterase inhibition, voltage-gated sodium channel disruption, and γ-aminobutyrate (GABA) inhibition may be involved (Maleki et al., 2023). However, evidence from epidemiological studies linking pesticide exposure and ASD suggests that the most relevant scenario is that of combined exposure to multiple pesticides (Yang et al., 2023). Pesticide residues from the diet, assessed in the EARLI study (n = 256), were not found to be associated with autism-related outcomes in children at age three (Joyce et al., 2022). Nevertheless, the deleterious effects of these pesticides are observed in rodent models (Yang et al., 2023).

Numerous epidemiological data demonstrate that exposure to bacterial or viral infections (e.g, rubella, measles, polio, herpes simplex …) or protozoan parasites (e.g., *Toxoplasma gondii*) during fetal life increases the risk of developing ASD, particularly during specific developmental windows (e.g., viral infections in the first trimester of pregnancy and bacterial infections in the second trimester) (Cattane et al., 2020). Furthermore, studies have attempted to identify correlations between maternal infections (such as SARS-CoV-2 or HIV), neurodevelopmental disorders, and disruptions in the maternal microbiome. The findings suggest that early infections during pregnancy are associated with alterations in the maternal gut microbiota, which may influence the severity of the maternal immune response (Orchanian and Hsiao, 2025).

These various studies clearly highlight the importance of prenatal environmental factors, including diet, medication, and environmental toxics, in shaping the gut microbiota and influencing the potential risk of ASD. However, a significant gap remains between findings from animal models and human epidemiological data. Longitudinal and standardized studies with comprehensive microbiome profiling are essential to better understand these complex interactions and their implications for neurodevelopmental outcomes.

#### 3.2.2 Maternal endogenous factors

##### 3.2.2.1 Microbiota

**Fig. 3:**
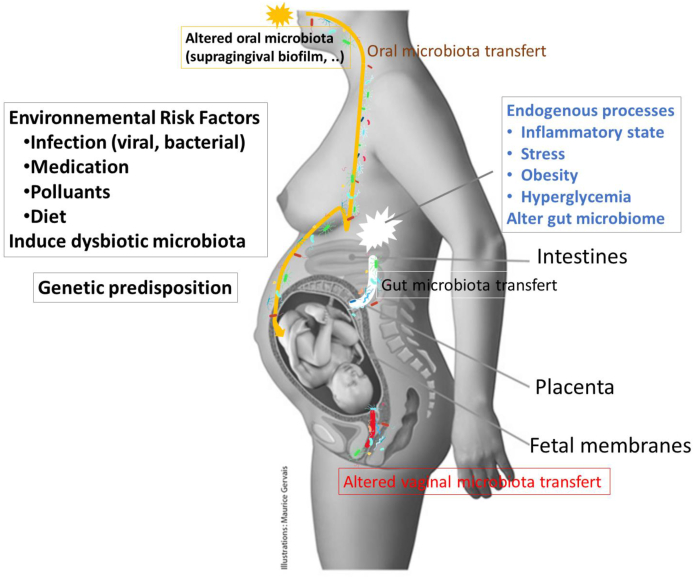
Potential pathways of fetal microbiota colonization and possible links between maternal impact and the occurrence of ASD in children, adapted from Kuperman et al. (Kuperman and Koren, 2016).

The maternal gut microbiome undergoes substantial changes during pregnancy by hormonal changes. As gestation progresses, intestinal diversity increases, particularly in Proteobacteria and Actinobacteria, with the most significant changes occurring during the second and third trimesters. The diverse microbiota could be the missing link between environmental insults during prenatal life and future neurodevelopmental disorders (Fig. 3).

Among 213 pregnant women, taxa from butyrate-producing families, such as *Lachnospiraceae* and *Ruminococcaceae*, were more abundant in mothers of neurotypical children (Dawson et al., 2021). In parallel, it has been suggested that maternal gut microbiota may be more predictive of early neurodevelopment than the infant's own microbiome (Sun et al., 2023).

**Fig. 4:**
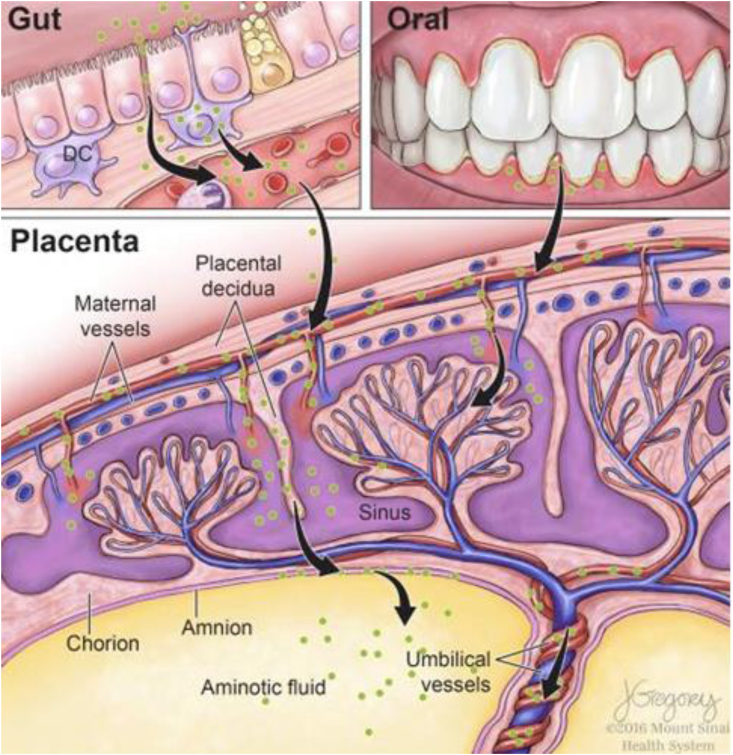
Suggested mechanisms of maternal transfer of bacteria to the fetus *in utero,* from Walker et al. (Walker et al., 2017).

Furthermore, some studies, largely controversial due to technical aspects of the protocol, have challenged the “sterile womb” dogma, as discussed above. The placenta is generally considered as a barrier to protect the fetus from microbial pathogens that invade the mother's bloodstream through the syncytiotrophoblast, cytotrophoblasts, and extravillous trophoblasts (Perez-Munoz et al., 2017). However, if these defenses are defective, bacteria already present in the endometrial mucosa or in the urogenital regions may be incorporated into the developing placental decidua. Bacteria transferred into the blood from other maternal microbiomes could reach the placenta and be transferred to the developing fetus via amniotic fluid and umbilical cord blood, as schematically depicted in Fig. 4.

The maternal oral microbiota could also influence fetal development. Indeed, dental injuries or surgeries and oral conditions causing inflammation (such as gingivitis) allow oral bacteria from salivary and sub-gingival microbiome communities to enter the circulatory system, and potentially colonizing the fetal environment (Fig. 3, Fig. 4).

Emerging human data describe how maternal microbes or their metabolites cross placental barriers, interact with fetal neurodevelopment, and shape long-term brain function and behavior.

##### 3.2.2.2 Term of pregnancy

Preterm infants exhibit lower intestinal microbial diversity compared to full-term infants, though this difference diminishes by age 2. A study in the USA has shown that children born significantly prematurely were 4.7 times more likely to develop ASD (Mir et al., 2020), as observed in the EPICure study (O'Reilly et al., 2021). Approximately half of preterm births (PTB) was linked to microbial etiologies, including pathologic changes to vaginal microbial diversity, with potential ethnic variation in susceptibility (Kumar et al., 2021). For example, Bresesti et al. suggested that maternal dysbiosis associated with PTB and ASD, characterized by a reduced microbiota alpha diversity and dysregulation of *Lactobacillus* and *Bifidobacterium*, could enhance the production of neuroactive molecules such as phenylalanine and GABA, thereby impacting early brain development (Bresesti et al., 2022).

##### 3.2.2.3 Placental dysregulation

Dysregulations of the placenta can increase the risk of ASD through various pathways, at different stages of pregnancy (Mir et al., 2020). In early pregnancy, inflammatory processes may disrupt placental angiogenesis and fetal neurogenesis. While the placental microbiome appears to resemble the oral (45 % similarity) rather than gut or vaginal microbiome (Walker et al., 2017), its role in neurodevelopmental disorders remains speculative due to limited supporting evidence (De Siena et al., 2021).

##### 3.2.2.4 Maternal stress

Elevated prenatal stress can affect the cognitive development though stress-related hormones, such as cortisol and placental CRH, and is correlated with ASD in offspring (Caparros-Gonzalez et al., 2021).

A study involving 459 mothers of children with ASD in Saudi Arabia observed a link between maternal stress during pregnancy and the severity of ASD in children, particularly in a population with a high prevalence of consanguineous marriages (Alamoudi et al., 2023). These data were obtained from a self-administered questionnaire designed to evaluate, retrospectively, the perception of maternal stress during pregnancy, administered after the children's diagnosis.

A cross-sectional study, involving 92 Australian children with ASD, concluded that MIA during pregnancy, but not maternal stress, was associated with a risk of ASD (Vasileva et al., 2024). However, neither MIA nor maternal stress induced any long-term alterations in the children's gut microbiome, which starkly contrasts with data from preclinical studies.

There is no single gestational period of specific vulnerability; the effects of prenatal stress vary depending on the gestational age and, consequently, on the developmental stage of specific brain regions and circuits, as well as the cause of stress and the state of the immune system (Van den Bergh et al., 2020). Other studies did not entirely support these findings, placing more emphasis on the pregnancy period (1st and 3rd trimesters) and the source of stress as being more impactful on the occurrence of neurodevelopmental disorders and the microbiota rather than stress itself (Cattane et al., 2020).

The various endogenous maternal conditions may influence ASD risk through immune, microbial, and hormonal pathways, though, evidence varies in strength and requires further longitudinal research to clarify these effects.

## 4 Perspectives

As mentioned in 2024 during an international meeting regarding the relationship between nutrition and ASD, research should consider the impacts of both maternal and paternal nutrition, as well as the maternal oral, vaginal, and gut microbiomes, to identify potential biomarkers for ASD risks (Maitin-Shepard et al., 2024). Despite its critical importance, the maternal – placenta - fetal brain remains insufficiently explored in ASD research.

### 4.1 Perspectives through regulating the gut microbiota

**Fig. 5:**
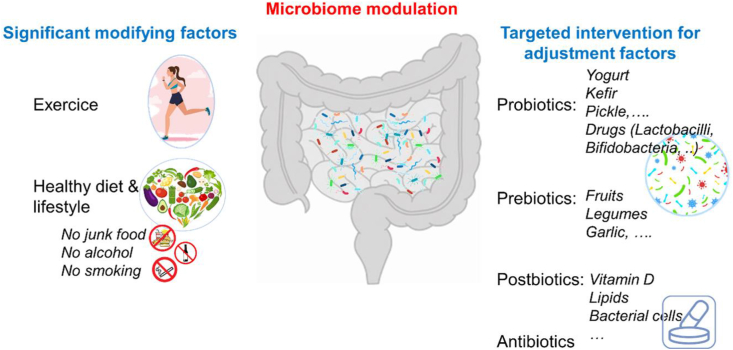
Interventions to modulate the gut microbiome.

Reversing potential dysbiosis, which needs to be characterized first, a point not extensively developed in the therapeutic framework, could be achieved through four approaches: pre-/pro-biotics, dietary interventions, antibiotics, and fecal microbiota transplantation (FMT) (Fig. 5). These interventions could reduce the ASD-related risks, especially for women with obesity, hypertension, or diabetes.

**Probiotics** are defined as living microorganisms, preferably of human origin. The main probiotics found in current commercial preparations include various species of *Lactobacillus* (e.g. *, casei, fermentum, gasseri, johnsonii, paracasei, plantarum, rhamnosus,* and *salivarius*) and *Bifidobacterium* (e.g., *adolescentis, animalis, bifidum, breve,* and *longum*) (Das et al., 2022). Probiotics are capable of stabilizing the mucosal barrier by increasing mucin expression, reducing bacterial overgrowth, stimulating mucosal immunity (secretory IgA), and synthesizing antioxidant metabolites. Probiotic administration of *Bacteroides fragilis* has been shown to correct intestinal permeability in offspring of immune-activated pregnant rodents (Golbaghi et al., 2024). The mechanism of *B. fragilis* action involves the production of capsular polysaccharide A (PSA) in the intestine, which increases SCFA production, IL-22 expression, and regulatory T cells, leading to partial correction of TJs expression (Sofi et al., 2021). But, newborn male mice treated with an overabundance of *B. fragilis* at birth have displayed social behavior dysfunction, increased repetitive behaviors, and gene expression dysregulation in the prefrontal cortex, while female mice did not show behavioral deficits (Carmel et al., 2023). This suggests that an overabundance of *Bacteroides*, in early life may have functional consequences for individuals with ASD.

A meta-analysis based on 14 studies with children with ASD, showed no significant differences in behavioral problems and symptom severity with probiotics compared to placebo (Tan et al., 2021). Only four non-randomized studies showed improvements in the overall severity of ASD (Srikantha and Mohajeri, 2019). Most studies (n = 10) assessed the gastrointestinal beneficial effects of pre- and synbiotics.

To prevent maternal separation-induced autistic-like behaviors in offspring, a prenatal probiotic intake (*Bifidobacterium longum* strain) was administrated to pregnant mice (Qing et al., 2025). The resilience against stress-induced gut pathogenic microbes and Th17-mediated peripheral inflammation inhibited hypermyelination and neuroinflammation linked to systemic inflammation. This strain can reshape the offspring's gut microbiome through vertical mother-infant transmission, in a preventive action (Qing et al., 2025).

Another approach to regulating microbiome dysbiosis is the use of prebiotics. **Prebiotics** include non-digestible fibers that are fermented by certain colon bacteria, promoting the growth of specific beneficial microbes and so, the production of SCFAs, especially, acetate and butyrate (Codagnone et al., 2019). The most studied prebiotics used are fructo- and galacto-oligosaccharide, lactulose, and inulin. Prebiotics show good tolerability and few adverse effects. Furthermore, a Japanese study (76 207 mother-infant pairs) has shown that maternal dietary fiber deficiencies during in mid-pregnancy might increase the risk of neurodevelopmental delay in offspring (Miyake et al., 2023).

But, these findings should not encourage the routine use of pre/probiotics, as they may create false hope (Tan et al., 2021). Therefore, it would be intriguing to analyze the intestinal microbiome of pregnant women and, if dysbiosis is clearly identified, a point not extensively developed in the therapeutic framework, it could be helpful to administer regulatory probiotics, well selected, during neurodevelopment.

Regarding MIA and microglial dysfunction during neurodevelopment, several therapeutic options could be considered, such as immunomodulators, luteolin, minocycline, suramin, and vitamin D.

Developmental **vitamin D** (DVD) deficiency is a well-established epidemiological risk factor for ASD. Furthermore, alterations to gut physiology have been described in a rat model of DVD-deficiency (Tamang et al., 2023). In a mouse model showing aberrations in placental Cyp11a1 expression, a key factor in steroid hormone synthesis, neurodevelopmental disorders in offspring were observed. Prenatal vitamin D3 (25(OH)D) supplementation (an active form of vitamin D) ameliorated the resulting neurobehavioral and neuroinflammatory derangements (Yin et al., 2024). Similarly, in a MIA mouse model, although prenatal administration of 25(OH)D abolished ASD-related behavioral deficits in the pups, but, it had no effect on pro-inflammatory cytokine levels in juveniles or fetal brains (Vuillermot et al., 2017). Human studies are mixed: In a Danish study involving 623 pregnant women (taking either a daily vitamin D3 supplementation of 2400 IU or a placebo), higher maternal 25(OH)D levels were associated with a decreased risk of ASD and a lower autistic symptom load (Aagaard et al., 2024). However, high-dose vitamin D3 supplementation during pregnancy had no effect on the risk of autism. A study in China (1321 children with ASD and 1200 typically developing children under 7 years old) analyzed the association between maternal vitamin D and multivitamin supplementation during pregnancy and the risk of ASD in offspring (Qi et al., 2024). The questionnaires did not include information about dosage, initiation time, frequency, or duration, nor did they specify the multivitamin supplementation. The authors found that maternal vitamin D and multivitamin supplementation during pregnancy were both significantly associated with a decreased risk of ASD in offspring. However, maternal vitamin D supplementation was not associated with symptoms or developmental quotients in children with ASD. Furthermore, a meta-analysis showed a small, but non-significant, trend suggesting a positive association between higher maternal vitamin D levels and offspring cognitive, language, motor, and social-emotional development (Varthaliti et al., 2025). The authors concluded that more studies are needed, considering the confounding factors contributing to inconsistencies, including supplementation protocols, genetic variations, and assessment methodologies, before confirming, or not, the role of maternal vitamin D in fetal neurodevelopment.

**Postbiotics** (nonviable bacterial products or metabolic byproducts from probiotic microorganisms) could also reduce the risk of developing ASD. Studies have described that prenatal administration of sodium butyrate (one of the SCFAs) improved neurodevelopmental outcomes of offspring in animal models of ASD (Cristiano et al., 2022). However, lower serum levels of acetate, butyrate, and isobutyrate, especially during the first trimester of pregnancy, were associated with better language and psychomotor development and, in the case of butyrate, better behavioral intensity in infants (Hernández-Martínez et al., 2022).

### 4.2 Other microbiota (vaginal, oral and paternal microbiota)

Other microbiota may impact fetal development, including those from the oral cavity, skin, vagina, and possibly the placenta (Fig. 2, Fig. 3). The placenta and amniotic fluid have low bacterial abundances (composed of commensal organisms from the *Firmicutes, Tenericutes, Proteobacteria, Bacteroidetes*, and *Fusobacteria* phyla), although this has been contested by the hypothesis that samples are sensitive to contamination (Lauder et al., 2016).

During a healthy pregnancy, the placenta generates estradiol and estriol, leading to increased *Lactobacillus* levels and a low vaginal pH, thus protecting both mother and fetus against infections. According to some studies, the newborn's intestinal microbiota would resemble the mother's postnatal **vaginal microbiota**. A difference in the gut microbiota has been highlighted depending on whether children are born vaginally or via cesarean section, which resulted in lower intestinal microbial diversity. In a meta-analysis of 5 million births across five countries, cesarean section was associated with a modestly increased risk of ASD from gestational weeks 36–42 compared to vaginal delivery (Yip et al., 2017).

To prevent the harmful effects that can result from vaginal dysbiosis, the use of probiotics is emerging as a promising strategy to improve vaginal health, reduce potential complications involving the mother's immune system, and support the establishment of the infant's microbiome. The most commonly used probiotics to treat bacterial vaginosis are *Lactobacilli* strains such as *L. reuteri RC-14, L. fermentum, L. gasseri, L. rhamnosus, L. acidophilus, L. crispatus, L. casei,* and *L. salivarius* (Dibo et al., 2022).

The **oral microbiome** has gained more and more interest regarding the systemic health. Comprising more than 700 bacterial species, the composition of the saliva microbiota undergoes slight changes from preconception to late pregnancy, with an increase in pathogens in saliva samples during pregnancy. This is due to elevated sex hormone levels, which leads to increased oral vascular permeability. During the first six months after birth, 85 % of the oral microbiota in infants resembled that of their mothers (Kaan et al., 2021).

Some studies have highlighted significant differences in the distribution of the oral microbiome between children with ASD and neurotypical children. However, selective eating, which leads to nutritional deficiencies, along with complicated oral health in children with ASD, could be part of the explanation for the divergence in the oral microbiome between children with ASD and controls (D'Angelo et al., 2025).

However, oral microbiota, from children with ASD, were administered into an antibiotic-mediated microbiota-depleted mouse model, leading to, ASD-like behaviors, differences in microbial community structures, and altered neurosignaling activities in the recipient mice (Qiao et al., 2022). This could highlight the mouth-microbiome-brain connection in the development of neuropathology. Additionally, a study involving 224 pregnant women, in their second-trimester, found that high trait anxiety induced higher richness of oral alpha diversity, and differences in beta diversity, reflecting alterations in microbe composition, with possible mechanisms including stress-induced pro-inflammatory oral cytokines impinging on the brain (Alex et al., 2024). Probiotic treatments might benefit for mothers with psychological distress.

However, to date, clinical studies in pregnant women are needed to uncover potential correlations between dysbiosis of the oral microbiota (oral infections, stress, …) and the risks of developing autistic disorders in offspring.

Finally, the **paternal microbiome** is an emerging paradigm highlighting the influence of paternal environmental exposures, including diet and lifestyle, on offspring health (Lo and Easley, 2025). For example, perturbations to the gut microbiota of prospective mice fathers increase the likelihood of their offspring presenting with severe growth restriction and premature mortality (Argaw-Denboba et al., 2024). This effect is linked to a dynamic response in the male reproductive system, including impaired leptin signaling, altered testicular metabolite profiles, and remapped small RNA payloads in sperm. Dysbiotic fathers could trigger an elevated risk of placental dysfunction.

## 5 Limitations

A key issue is that there are few epidemiological studies on ASD that focus on the preconception, and pregnancy. The links between ASD and prenatal factors are primarily retrospective, making it difficult to trace prenatal data. The characterization of different microbiomes is not yet possible in routine clinical practice, and the definition of a “standard” healthy microbiome has not been established. However, any characterization of dysbiosis would be a significant step toward therapeutic intervention using pro/pre/postbiotics.

Overcoming the considerable gaps in transferring results from reductionist preclinical models to human clinical practice is crucial.

There is also a lack of research into paternal nutrition and gut microbiota. Much remains to be done in this area, particularly regarding preventive aspects.

## 6 Conclusion

The exposome, encompassing both external and endogenous maternal factors, plays a critical role in shaping the fetal environment and influencing long-term health outcomes in the offspring. Maternal influences, such as diet, stress, toxins, …. may contribute to a dysregulated microbiome, which in turn can increase gut permeability, elevate proinflammatory cytokine levels, and disrupt the production of SCFAs, collectively promoting systemic inflammation and an altered immune response. ASD are complex neurodevelopmental conditions, where microbiota may influence the interaction between environmental factors and physiological balance. Dysbiosis in the maternal microbiota disrupts fetal brain development and may contribute to autism-related phenotypes. Key microbiomes, including those in the gut, oral cavity, and vagina, regulate crucial processes like immune activation and inflammatory responses during neurodevelopment. The ASD ethology may involve both human and microbial genetics, shaped by genome-environment interactions. Studying the maternal microbiota could help identify early risk factors and lead to targeted preventive strategies, such as dietary changes. While this could be implemented for women at risk, caution is needed to avoid false hope.

## CRediT authorship contribution statement

**Sylvie Mavel:** Writing – review & editing, Writing – original draft, Visualization, Conceptualization. **Léa Pellé:** Writing – original draft, Conceptualization. **Christian R. Andres:** Writing – review & editing, Writing – original draft.

## Funding

The work was supported by the French “Institut National de la Santé et de la Recherche” INSERM and the 10.13039/501100007526University of Tours.

## Declaration of competing interest

The authors declare that they have no known competing financial interests or personal relationships that could have appeared to influence the work reported in this paper.

## Data Availability

No data was used for the research described in the article.
